# Biliary Epithelial Apoptosis, Autophagy, and Senescence in Primary Biliary Cirrhosis

**DOI:** 10.1155/2010/205128

**Published:** 2010-11-04

**Authors:** Motoko Sasaki, Yasuni Nakanuma

**Affiliations:** Department of Human Pathology, Kanazawa University Graduate School of Medicine, Kanazawa 920-8640, Japan

## Abstract

Primary biliary cirrhosis (PBC) is a chronic cholestatic liver disease characterized serologically by the high prevalence of anti-mitochondrial autoantibodies (AMAs) and histologically by the cholangitis of small bile ducts, eventually followed by extensive loss of the small bile duct. An autoimmune pathogenesis is suggested by clinical and experimental studies, but there remain issues regarding the etiology, the significance of AMAs in the pathogenesis of bile duct lesions, and so on. The unique properties of apoptosis in biliary epithelial cells (BECs), in which there is exposure of autoantigen to the effectors of the immune system, are proposed to be a cause of bile duct lesions in PBC. Recent progress disclosed that cellular senescence and autophagy are involved in bile duct lesions in PBC. Senescent BECs may modulate the periductal microenvironment by expressing senescence-associated secretory phenotypes, including various chemokines, and contribute to the pathogenesis of bile duct lesions in PBC.

## 1. Introduction

Primary biliary cirrhosis (PBC) is a chronic, progressive cholestatic liver disease in which autoimmune pathogenesis is suggested [1–4]. It usually affects middle-aged women [1, 5] and often leads to liver failure and liver transplantation [2, 3]. PBC is characterized histologically as cholangitis of small bile ducts (chronic nonsuppurative destructive cholangitis; CNSDC) eventually followed by the extensive loss of small bile ducts [2, 3, 6]. Biliary epithelial cells (BECs) are thought to be the major target of injury in PBC. PBC is serologically characterized by the presence of increased levels of immunoglobulin M (IgM), a high titer of serum antimitochondrial autoantibodies (AMAs), and, in some patients, PBC-specific antinuclear antibodies (ANAs) [1, 2, 7–10]. AMAs are present in about 95% of PBC cases, with disease specificity close to 100%, and are therefore considered the serological hallmark of the disease. The major epitope site for both the B-cell and CD4 and CD8 T-cell response is an inner lipoyl domain of the E2 component of pyruvate dehydrogenase (PDC-E2) [10–13]. 

There have been many studies on the immunopathological features [10–13], genetic factors [14–17], and environmental factors [5, 18–20], including infectious agents and xenobiotics. The most accepted hypothesis states that PBC results from an environmental insult on a genetically susceptible background. In this scenario, adaptive immunity, both humoral and cellular (CD4 and CD8 T cells), and innate immunity have been proposed as coplayers in immune-mediated liver damage; however, there remain many mysteries in the etiology and pathogenesis of PBC; in particular, the significance of AMAs and autoantigen-specific T-cell response in the pathogenesis of bile duct lesions remains unknown. One hypothesis proposed that a BEC-specific autoimmune reaction, possibly causing bile duct lesions, is a unique property of apoptosis in biliary epithelial cells (BECs), in which there is exposure of autoantigen to the effectors of the immune system [4, 21–23]. In addition, our recent studies disclosed that cellular senescence and autophagy are involved in the bile duct lesion in PBC [24–28]. These two novel cellular processes may be related to the immunopathology of BECs together with AMAs in PBC. At least, it is likely that senescent BECs modulate the periductal microenvironment by expressing senescence-associated secretory phenotypes (SASPs) including various chemokines and contribute to the pathogenesis of bile duct lesions in PBC [29]. In this paper, we will focus on studies addressing the features of BECs in PBC and its possible involvement in the progression of diseases.

## 2. Autoantibodies in PBC

### 2.1. AMAs

AMAs are present in about 95% of PBC cases, with disease specificity close to 100%, and are therefore considered the serological hallmark of the disease. The targets of AMAs are members of the 2-oxoacid dehydrogenase complex (2-OADC), including the E2 subunit of pyruvate dehydrogenase complex (PDC-E2), the E2 subunit of branched chain 2-oxoacid dehydrogenase complex (BCOADC-E2), the E2 subunit of 2-oxoglutarate dehydrogenase complex (OGDC-E2), and dihydrolipoamide dehydrogenase binding protein (E3BP), which is a component of the PDC complex [1, 7–10]. The most common AMAs reactivity is with PDC-E2; whereas some patients have AMAs reacting with PDC-E2 alone, most have reactivity also with OGDC-E2 or BCOADC-E2; reactivity to BCOADC-E2 or OGDC-E2 alone is less common. It is located in the mitochondrial matrix, associated with the inner membrane, and catalyzes the oxidative decarboxylation of various *α*- ketoacid substrates. The major epitope site of both B-cell and CD4 and CD8 T-cell responses is the inner lipoyl domain of the E2 component of pyruvate dehydrogenase (PDC-E2) [10–13]. These domains contain ETDKA, ETDK(T), and (GlnS)DKA with lipoic acid covalently bound to the *ε* group of lysine (K). In addition to sera, AMAs, especially the IgA isotype, and the autoantigens, PDC-E2, OGDC-E2, and BCOADC-E2, were detected frequently in the bile of patients with PBC [30].

Although AMAs are detected in approximately 95% of patients, their direct pathogenetic role is still poorly understood. A minor population of patients is AMA-negative, and these AMA-negative cases manifest similar histological features and disease progression [31]. In autoimmune disease, the reduction of disease-specific autoantibody titers correlates with disease amelioration; this criterion is not suitable for PBC, in which there is no correlation between the pattern or titer of AMAs and the progression or severity of diseases [4, 32]. Furthermore, most autoimmune diseases are responsive to immunosuppressive therapy, while no such agent has proven effective for PBC [33]. Further, in the autoimmunity paradigm, experimental immunization with disease-specific autoantigens rarely reproduces a model disease.

### 2.2. ANA

In PBC, there are highly disease-specific and antigen-specific antinuclear activities directed as a variety of antigens, including centromere, nucleoporins gp210 and p62, and Sp100 [34–36]. Gp210 is a 210-kd transmembrane glycoprotein believed to be involved in the attachment of pore complex constituents in the nuclear membrane. These antibodies are found in about 25% (between 10% and 40%) of patients with AMA-positive PBC and up to 50% of those with AMA-negative PBC; the specificity for PBC, when detected by immunoblotting, is over 99% [34]. p62 is another nuclear pore glycoprotein specific to PBC and is present in about 25% of patients with PBC [35], although patients with anti-gp210 and p62 appear to be mutually exclusive. 

In contrast to AMAs, there is a significant correlation of disease severity and clinical types with antibodies to gp210 and centromere [36]. Nakamura et al. reported that positive anti-gp210 antibodies were a siginificant risk factor for the hepatic failure type of progress, whereas positive anti-centromere antibodies were a significant risk factor for portal hypertension-type progression [36]. Histologically, positive anti-gp210 antibodies were most significantly associated with more severe interface hepatitis and lobular inflammation, whereas positive anti-centromere antibodies were most significantly associated with a more severe ductular reaction [36]. The pathological role of these antibodies, however, has been poorly investigated so far, and this clearly warrants further research.

## 3. Biliary Epithelial Lesions in PBC

### 3.1. Histology of PBC

The bile duct damage characteristic of early PBC mainly affects the septal and larger interlobular bile ducts, while the smaller interlobular ducts remain intact until later. The term chronic nonsuppurative destructive cholangitis (CNSDC) more accurately describes the initial lesions in PBC [2, 3, 6, 37]. The epithelium of the affected ducts becomes irregular and infiltrated with lymphocytes. The basement membrane becomes disrupted, and the duct may rupture. An inflammatory infiltrate is seen around or to one side of the duct. The denser parts of this infiltrate are mainly composed of lymphocytes, which may form aggregates of follicles with germinal centers. Elsewhere, there is a mixture of plasma cells, often abundant, eosinophils, and neutrophils. Granulomas are present in many patients, although they are not necessarily seen [37]. They take a variety of forms, ranging from well-defined granulomas, like those of sarcoidosis or tuberculosis, to small focal collections of histiocytoid cells. 

The affected bile ducts eventually disappear from the liver, and chronic cholestatic features develop gradually. At the same time, hepatitis activity of varying degrees is frequently imposed on the liver. Chronic cholangitis activity and hepatitis activity in various combinations may be responsible for progressive hepatocellular damage and fibrosis, and cirrhosis and hepatic failure finally develop. We proposed a new histological staging and grading system of PBC for comprehensive analysis of the histological progression of PBC (staging) toward extensive bile duct loss, chronic cholestasis, and cirrhosis, and also the immune-mediated necroinflammatory activity of small bile ducts and hepatocytes [38].

## 4. Immunopathology of PBC

Mechanisms involved in the disruption of the biliary epithelium in PBC, especially the association between AMAs and bile duct damage, remain poorly understood. Several mechanisms have been proposed regarding immune-mediated bile duct damage in PBC, including (1) T-cell-mediated cytotoxicity and (2) interaction of the IgA class of AMAs and mitochondrial autoantigens in BECs during intracellular transport, resulting in cytotoxicity. In the autoimmune pathology of PBC, it remains controversial whether BECs are innocent victims or active participants [4, 39]. BECs may be active participants rather than innocent victims in the autoimmune pathology of PBC [4], due to the anomaly of apoptosis by which a lack of glutathionylation exposes PDC-E2 in BECs to autoimmune effector agents [21–23, 40]. Such properties may include unique processes of apoptosis, mechanisms related to mitochondrial autophagy, the presence of poly Ig receptors and, especially, in the case of BECs, a capacity to elicit intense mucosal responses [41]. In contrast, Shimoda et al. proposed that BECs are in fact innocent victims of autoimmune injury and that the adaptive immune response is critical in PBC [39]. They reported that BECs isolated from PBC patients and controls express similar levels of Toll-like receptor subtypes, CD40 and HLA-DR [39]. Interestingly, however, BEC-expressed chemokines elicit enhanced transmigration of PBC liver-infiltrating mononuclear cells compared with controls [39].

## 5. Altered Features of BECs in PBC

As BEC is thought to be a major target of immune-mediated attack, a number of studies have focused on the changes of BECs in PBC in comparison with other hepatobiliary diseases [4, 37, 42]. Because of the specificity of interlobular bile duct damage in PBC patients, it is logical to hypothesize that this particular segment of bile ducts expresses a specific antigenic molecule presented to the immune system. In addition, the heterogeneity of BECs related to the size of the bile duct was noted to address the question of why only small bile ducts, but not large bile ducts, are affected in PBC. Several studies have reported the altered characteristics of BECs in PBC. Most of these changes are related to epithelial damage and suggested to be reactive changes irrespective of disease [5, 42–47]. There have been few changes in BECs that are truly specific to PBC. Recently, we have reported that BECs in damaged small bile ducts show features of cellular senescence and autophagy in PBC and speculated that the cellular senescence may be involved in the pathogenesis of progressive bile duct loss in PBC [24–28].

### 5.1. Immune Adhesion Molecules

Several studies suggest that BEC is an antigenically distinct expressing molecule associated with the immune recognition of targets [42]. These include MHC class II (HLA-DR) and ICAM-1, which are critical for the interaction with lymphocytes and are upregulated in PBC [48–50], although these molecules are commonly induced in liver disease and are not specific to PBC. MHC class-II (HLA-DR) was newly expressed during the intermediate stage of PBC, but early in the disease HLA-DR expression on BEC is not detected or is present in only a few ducts [49]. Furthermore, BECs also express high levels of several adhesion molecules, including ICAM-1 and LFA-3, which are important for mediating adhesion to lymphocytes [48, 50].

### 5.2. Inflammatory Cytokines and Chemokines

#### 5.2.1. IL-6 and TNF-*α*

BECs overexpress IL-6 and TNF-*α* in PBC and, to a lesser degree, in other hepatobiliary diseases [47]. TNF receptor and IL-6 receptor were detected on these damaged bile ducts, suggesting an autocrine effect [47]. The increased expression of IL-6 and TNF-*α* could first affect the proliferation, maturation, and regulation of B-cell and T-cell lineages infiltrating around bile ducts [51]. IL-6 promotes the terminal differentiation of B cells and immunoglobulin secretion. TNF-*α* may induce the expression of adhesion molecules and various HLA-DR antigens on bile ducts and may also increase the cytotoxic activities of T cells. IL-6 may be responsible for biliary epithelial proliferation via autocrine effects [52], and TNF-*α* may be involved in biliary epithelial cell damage [53]. TNF-*α* is also known to disturb the barrier function of bile ducts, which may lead to the leakage of toxic substances in bile, a local inflammatory process, and cholangitis [54].

#### 5.2.2. Chemokines

Previous reports, including those from our group, have reported the upregulation of several chemokines, that is, CCL2/MCP-1, CX3CL1/fractalkine in BECs in PBC [29, 55–57]. It is conceivable that BEC-expressed chemokines participate in the accumulation and migration of various inflammatory cells, forming bile duct lesions in PBC. Our recent study [29] suggests that some of these chemokines may belong to senescence-associated secretory phenotypes (SASP) secreted by senescent BECs, as will be discussed below.

### 5.3. MUC Mucins and Trefoil Factors

Mucins and trefoil factors (TFFs) play a role in the protection of mucosa forming a mucus barrier. The intrahepatic biliary tree shows the site-characteristic expression of mucins and TFFs [58, 59]. BECs express physiologically a glycosylated form of MUC1 detected by anti-EMA antibody in intrahepatic small bile ducts. MUC4 and MUC5B mucins are also expressed focally and weakly in BECs in normal small bile ducts. 

#### 5.3.1. MUC1

In the normal liver, MUC1 mucin of the unglycosylated form detected by DF3 is infrequently and focally expressed in BECs in small bile ducts. In contrast, MUC1 mucin of the unglycosylated form is frequently and strongly positive on the luminal surface of BECs in small bile ducts in PBC and chronic viral hepatitis (CVH) [60]. In particular, a high level of MUC1 mucin of the unglycosylated type is expressed in BECs in small bile ducts involved in CNSDC in PBC and hepatitic bile duct lesion in CVH [60]. The frequent expression of MUC1 mucin of the unglycosylated type may therefore be a reactive change to injuries in BECs [60].

#### 5.3.2. MUC6

MUC6 mucin is focally and weakly expressed in small bile ducts in normal livers [43]. The expression of MUC6 mucin is increased in small bile ducts in CVH and, to a lesser degree, in other hepatobiliary diseases, including PBC. The extent of MUC6 expression in bile ductules is in parallel to the degree of active inflammation in CVH [43]; therefore, it is conceivable that MUC6 mucin is up-regulated in inflammatory conditions and may play a role as a cytoprotective agent [43].

#### 5.3.3. TFFs

The intrahepatic biliary tree shows the site-characteristic expression and induction of TFF1, 2, 3 and DMBT1, a putative receptor of TFFs. In large bile ducts, TFF1 and 3 are constitutively expressed and increased in pathologic bile ducts [45, 61, 62]. In small bile ducts, TFF2/DMBT1 is induced in damaged ducts, irrespective of the etiology. For example, the augmented expression of TFF2/DMBT1 is observed in BECs involved in CNSDC in PBC and hepatitic bile duct lesions in CVH. The expressions of TFF1 and 3 are also increased in BECs involved in CNSDC in PBC [45]. TFF3 expression is regulated by IL-6 via a signal transducer and activator of the transcription (STAT3) signaling pathway, and TFF3 contributes to BEC migration [63].

## 6. Cellular Processes Involved in PBC: Apoptosis, Autophagy, and Cellular Senescence

**Figure 1:**
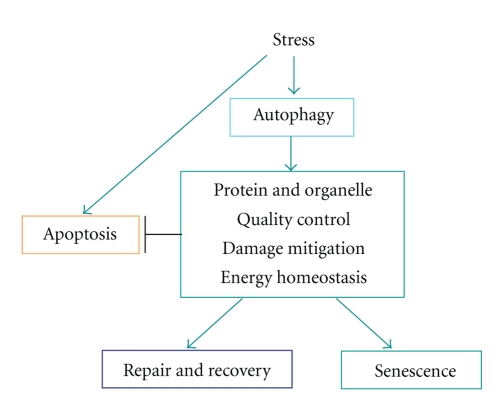
Cellular processes: apoptosis, autophagy, and cellular senescence are distinct cellular response to stress. Autophagy and apoptosis are induced in response to awide variety of stresses. There are many purposes for autophagy that include the quality control of protein and organelle, damage mitigation, and energy homeostasis. Stressed cells activate autophagy, which prevents damage and maintains metabolism though lysosomal turnover of cellular components. Autophagy can facilitate senescence or limit damage and delay apoptosis to allow recovery of normal cell function.

Apoptosis, autophagy, and cellular senescence are distinct cellular responses to stress, correlating with each other (Figure 1) [64]. An appropriate cellular stress response is critical for maintaining tissue integrity and function and for preventing diseases [64]. Cells responding to stress with adaptation, repair, and recovery are diverted into irreversible cell cycle exit (senescence) or are eliminated through programmed cell death (apoptosis) [64]. Autophagy, which literally means “self-eating,” is an evolutionarily conserved process that results from various cellular stresses such as nutrient damage and activation of the endoplasmic reticulum stress pathway [65, 66]. Autophagy can enable adaptation to stress through the degradation of cellular proteins and organelles to suppress damage, maintain metabolism, and promote cellular viability and fitness [64–67]. These cell fate decisions are critical to dealing with the emergence of damaged and potentially dangerous cells that can cause cancer. Cellular senescence is a state of stable cell arrest with active metabolism. Similar to apoptosis, senescence can be a failsafe program against a variety of cellular insults. In contrast to apoptosis, however, in which the cytotoxic signals converge to a common mechanism, senescence is typically a delayed stress response involving multiple effector mechanisms. Autophagy can delay apoptosis and is induced during the process of senescence which it facilitates [68].

Apoptosis in PBC has been studied most vigorously as an effector system of T-cell-mediated cell injury. Apoptosis of BECs and the altered expression of apoptosis-related molecules have been reported in bile duct lesion [69, 70], but immune-mediated injuries of BECs have not been fully clarified. Unique properties of apoptosis in BECs may play a role in the immune tolerance breakdown [4, 21–23]. In addition, our recent studies disclosed that cellular senescence and autophagy are involved in bile duct lesions in PBC [24–28]. These novel cellular processes, autophagy and cellular senescence, may be related to the immunopathology of BECs together with AMAs in PBC.

### 6.1. Apoptosis and PBC

#### 6.1.1. Molecules Related to Apoptosis

There is evidence that BECs undergo apoptosis in PBC for a study using in situ nick-end labeling methods to detect the DNA fragmentation of apoptosis, although the mechanisms responsible are not clear [69]. However, it appears difficult to distinguish apoptosis, and necrotic DNA fragmentation did not necessarily occur. Studies have shown the increased expression of perforin and granzymes in PBC, and Fas (CD95) is upregulated on the biliary epithelial cell membrane, so it is possible that both of these pathways are involved [70].

#### 6.1.2. Unique Features of BECs during Apoptosis

PBC bile duct cells manifest unique features during apoptosis while coculture experiments do not support a direct role for these cells in determining their immune-mediated injury. Odin et al. first demonstrated that PDC-E2 remains intact and retains its immunogenicity in BECs during apoptosis because of a cell-specific lack of glutathionylation of BECs [23]. The intact PDC-E2 in apoptotic fragments could be taken up by local antigen-presenting cells and transferred to regional lymph nodes for the priming of cognate T cells. Lleo et al. reported that BECs translocate intact PDC-E2 immunologically to apoptotic bodies, creating an apotope [21, 22]. They also demonstrated inflammatory cytokine production in the presence of a unique triad of BEC apotopes, and macrophages from patients with PBC and AMAs [21]. These studies may provide insights into why autoimmune damage is primarily confined to BECs in small bile ducts [21]. Allina et al. reported that apoptotic BECs were phagocytosed by BECs in PBC, and this may consequently provide an exogenous source of autoantigens in BECs [40].

### 6.2. Autophagy and PBC

Autophagy is an evolutionarily conserved process that results from various cellular stresses such as nutrient damage and activation of the endoplasmic reticulum stress pathway [65–67]. Autophagy can enable adaptation to stress through the degradation of cellular proteins and organelles to suppress damage, maintain metabolism, and promote cellular viability and fitness [65–67]. Three types of autophagy— macroautophagy, microautophagy, and chaperone-mediated autophagy— have been classified and macroautophagy is the major type [65–67]. It is now clear that macroautophagy (hereafter referred to as autophagy) is important for many physiological and pathological processes. During stresses such as nutrient deprivation or mitochondrial damage, autophagy is activated and organelles are sequestered in autophagosomes and digested by fusion with lysosomes to either generate energy or contain collateral damage such as induction of apoptosis due to damaged mitochondria. This evolutionarily conserved process is characterized by the formation of double-membrane cytosolic vesicles, autophagosomes, which sequester the cytoplasmic content and deliver it to lysosomes [71, 72]. Autophagy is often associated with acute metabolic changes and rapid protein replacement. Microtubule-associated protein-light chain 3*β* (LC3), a homologue of autophagy-related protein 8 (Apg8p), which is essential for autophagy and associated with autophagosome membranes after processing, is a widely used marker of autophagy [68, 73]. Recent studies disclosed that autophagy is induced during the process of senescence which it facilitates [68]. 

Autophagy may be a novel player in autoimmunity, that is, by MHC-class II presentation of cytosolic antigens and control of T-cell homeostasis [41, 74, 75]. Autophagy-related processing of self-proteins provides a source of immunostimulatory molecules and autoantigens [41, 74, 75]. 

#### 6.2.1. Biliary Epithelial Autophagy in PBC

**Figure 2:**
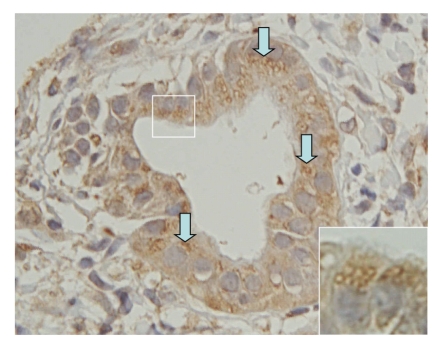
Biliary epithelial autophagy in PBC. The expression of autophagy marker LC3 was detected in intracytoplasmic vesicles (arrows) in BECs involved in damaged small bile ducts in PBC. Immunostaining for LC3. Original magnification, ×400 (inset, ×1000).

In our recent study, autophagy was specifically upregulated in damaged small bile ducts along with cellular senescence in PBC [28] (Figure 2). A representative autophagy marker, LC3, was characteristically expressed in cytoplasmic vesicles in the damaged small bile ducts in PBC [28]. Senescent markers p21^WAF1/Cip1^ and p16^INK4a^ were coexpressed with autophagic marker LC3 in damaged bile ducts in PBC [28]. The inhibition of autophagy suppressed cellular senescence in cultured cells [28]. These findings suggest that autophagy may mediate the process of biliary epithelial senescence and be involved in the pathogenesis of bile duct lesions in PBC. A major question related to this study is how autophagy/cellular senescence is related to the autoimmune etiology proposed for PBC. Autophagy of BECs may play a role in the immune tolerance breakdown of autoantigen: PDC-E2 in PBC, although this is only speculative at this moment. This important issue remains to be fully clarified.

### 6.3. Cellular Senescence in PBC

Cellular senescence is defined as a condition in which a cell no longer has the ability to proliferate. Senescent cells are irreversibly arrested at the G1 phase of the cell cycle and do not respond to various external stimuli but remain metabolically active. Senescent cells display several characteristics, including histological changes in vitro and in vivo [76, 77], shortened telomeres, increased expression of p16^INK4^ and p21^WAF1/Cip^, and increased activity of senescence-associated *β*-galactosidase (SA-*β*-gal) [78]. Cellular senescence can be triggered by multiple mechanisms, including telomere shortening, epigenetic derepression of the INK4a/ARF locus, and DNA damage [79]. Cellular senescence imposes a potent barrier to tumorigenesis and contributes to the cytotoxicity of certain anti-cancer agents [79, 80]. Interestingly, senescent cells have also been observed in certain aged or damaged tissues and have been suggested to limit cell depletion and the decline of tissue regeneration capacity with age [79]. Senescence may also act as part of a homeostatic mechanism to limit wound-healing responses following tissue damage [81]. Recent progress in the field of hepatology has disclosed that cellular senescence is involved in the pathophysiology of various chronic liver diseases [24–26, 81–84] and hepatocarcinogenesis [85, 86].

#### 6.3.1. Biliary Epithelial Senescence in PBC

**Figure 3:**
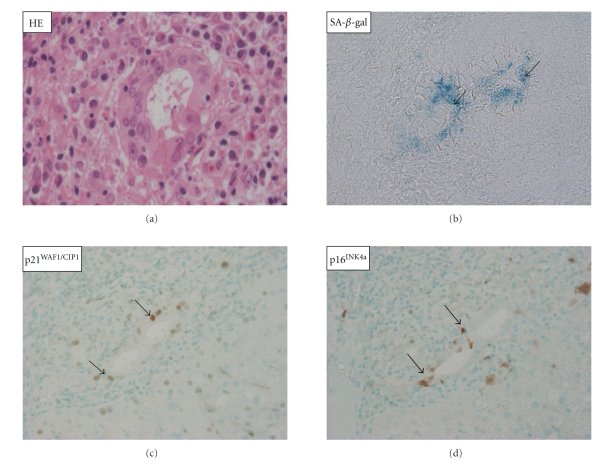
Biliary epithelial senescence in PBC. BECs in small bile ducts involved in CNSDC show histological features of senescence, such as cytoplasmic eosinophilia, cellular and nuclear enlargement, and uneven nuclear spacing (a). SA-*β*-gal activity is detected in BECs in PBC (b). Senescent markers, p21^WAF1/Cip1^ and p16^INK4a^, were expressed in BECs in damaged small bile ducts in PBC (c, d). Immunostaining for p21^WAF1/Cip1^ and p16^INK4a^. Original magnification, ×400.

We have reported the cellular senescence of BECs with shortened telomeres, the expression of SA-*β*-gal, and the augmented expression of p16^INK4a^ and p21^WAF1/Cip^ in damaged small bile ducts in PBC (Figure 3) and suggested that cellular senescence may be involved in the pathogenesis of progressive bile duct loss in PBC [24, 27]. The possible association of oxidative stress and decreased expression of polycomb group protein Bmi1 was suggested to be involved in the pathogenesis of cellular senescence in PBC [24–26]. Similar biliary epithelial senescence was reported in chronic liver allograft rejection, in which intrahepatic bile ducts are diminished [82]. 

There are many unanswered questions to clarify how senescent BECs relate to bile duct loss (ductopenia) in PBC. Senescent cells are known to contribute to impaired tissue integrity and persistent inflammation [87]. When cellular senescence occurs in injured BECs, these senescent cells are thought to remain *in situ* and not to be replaced by normal cells, although nonsenescent BECs proliferate in response to injury [88]. It is conceivable that impaired replacement and nonproliferative properties of senescent BECs make them prone to further injuries, accentuating inflammation by SASP, which is likely to be followed by bile duct loss in PBC. The fate of senescent BECs remains to be clarified: whether senescent BECs are removed by apoptosis, anoikis, or necrosis. Our previous studies reported that cellular senescence is frequently seen in bile ductular cells in a ductular reaction, which are thought to harbor hepatic stem/progenitor cells in PBC [24, 27]. This phenomenon may also be related to the impaired regeneration of BECs in small bile ducts in PBC.

#### 6.3.2. Senescence-Associated Secretory Phenotypes (SASPs)

Recent studies suggest that senescent cells play an important role in modulating the microenvironment by secreting biological active molecules, such as cytokines (IL-6, IL-1, and so on), chemokines (CXCL8/IL-8, CCL2/monocyte chemotactic protein-1 (MCP)-1), and so on), growth factors, and profibrogenic factors [89–93]. In fact, studies in humans with biliary disorders and in animal models of biliary fibrosis have shown that the ductal epithelium can express a number of profibrogenic and chemotactic proteins (e.g., IL-1, IL-6, CXCL8/IL-8, and CCL2/MCP-1), the latter capable of attracting and activating cells of both inflammatory and stellate cell lineage [39, 55, 56, 94]. These cytokines and chemokines may belong to SASPs [89–93].

#### 6.3.3. SASPs in PBC

**Figure 4:**
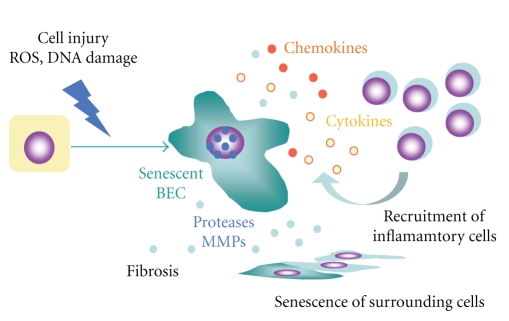
Possible regulation of microenvironment by senescent BECs expressing SASPs such as chemokines and cytokines in PBC. Senescent BECs may participate in modulation of the inflammatory microenvironment by recruiting monocytes and possibly other types of inflammatory cells, induction of senescence in surrounding cells, and progression of fibrosis via SASP.

Previous reports, including those from our group, have reported the upregulation of several cytokines and chemokines in damaged bile ducts in PBC as described above [39, 47, 56], and recent studies have disclosed that most of these factors are known to belong to SASPs [89–93]. Our recent study disclosed the involvement of senescent BECs in modulation of the inflammatory microenvironment around affected small bile ducts in PBC [29]. We examined the chemokine profiles in cultured senescent BECs and the chemotactic capacity and also the correlation between the expression of senescent features and chemokine profiles in human PBC livers by immunohistochemistry [29]. Senescent BECs induced by various stresses expressed a significantly higher level of chemokines. Senescent BECs significantly facilitated the migration of RAW264.7 cells, and neutralizing antibodies against CCL2 and CX3CX1 partially blocked the migration induced by senescent BECs. The expression of CCL2 and CX3CL1 was significantly higher in BECs in inflamed and damaged small bile ducts in PBC than in noninflamed bile ducts and control livers. The expression of CCL2 and CX3CL1 was colocalized with the expression of senescent markers. These findings suggest that senescent BECs may participate in modulation of the inflammatory microenvironment by recruiting monocytes and possibly other types of inflammatory cells via SASP (Figure 4).

Very little is known about the mechanism which initiates and maintains SASPs, including chemokines [79, 89–91]. Since several types of cellular stress, such as oxidative stress, DNA damage by etoposide, and serum deprivation, induced SASPs in senescent BECs in the present study, and it is plausible that these stresses may induce SASPs via a common mechanism in the senescent state. Similarly, SASPs in senescent BECs in PBC may contribute to the activation of the innate immune system around the injured bile ducts. Upregulation of several cytokines and chemokines has been reported in damaged bile ducts in PBC [39, 47, 56]. This study raised the possibility that most of such cytokine and chemokine profiles may be included in SASPs. Senescence is both regulated by and regulates the extracellular environment.

## 7. Summary

PBC, like most polygenic autoimmune diseases, clearly belongs to the “complex disease” category that is attributable to the combined effects of multiple environmental and behavioral influences, genetic elements, and perhaps chance. Mitochondrial autoantigens and B-cell and T-cell autoepitopes have been well characterized in PBC; however, the etiology and the relation between AMAs and bile duct lesions remain to be determined. We emphasized the features of BECs in bile duct lesions in PBC, in particular, the unique feature of apoptotic BECs which retain immunologically intact PDC-E2 and two novel cellular processes: autophagy and cellular senescence. Autophagy may be a promising cellular mechanism involved in the autoimmune mechanism together with apoptosis. Cellular senescence may be related to the immunopathology of BECs by the expression of SASPs in PBC. Further studies are warranted to completely disclose the pathogenesis of PBC.
